# Anti-Proliferative Effects of Lidocaine as an Autophagy Inducer in Bladder Cancer via Intravesical Instillation: In Vitro and Xenograft Mouse Model Experiments

**DOI:** 10.3390/cancers16071267

**Published:** 2024-03-24

**Authors:** Young Chul Yoo, Na-Young Kim, Seokyung Shin, Yunil Yang, Ji Hae Jun, Ju Eun Oh, Myoung Hwa Kim

**Affiliations:** 1 Department of Anesthesiology and Pain Medicine, Anesthesia and Pain Research Institute, Yonsei University College of Medicine, 50-1 Yonsei-ro, Seodaemun-gu, Seoul 03722, Republic of Korea; seaoyster@yuhs.ac (Y.C.Y.); knnyyy@yuhs.ac (N.-Y.K.); skshin@yuhs.ac (S.S.); 2 Department of Anesthesiology and Pain Medicine, Anesthesia and Pain Research Institute, Yonsei University College of Medicine, Gangnam Severance Hospital, Eonju-ro 211, Gangnam-gu, Seoul 06273, Republic of Korea; 2015191045@yuhs.ac; 3 Anesthesia and Pain Research Institute, Yonsei University College of Medicine, 50-1 Yonsei-ro, Seodaemun-gu, Seoul 03722, Republic of Korea; ilovepodo@yuhs.ac

**Keywords:** anti-tumor effect, bladder cancer, lidocaine, Nara Bladder Tumor No. 2

## Abstract

**Simple Summary:**

Our in vitro and in vivo experiments demonstrated that bladder tumor growth can be attenuated even with the intravesical administration of lidocaine as a single agent, and that the mechanism involves autophagy influx. These findings support the potential of intravesical lidocaine injection for clinical application. Further successful validation using human-derived bladder cancer cell lines and confirmation of the effectiveness of intravesical lidocaine administration in patients in clinical trials could help establish lidocaine as a novel adjuvant treatment for bladder cancer.

**Abstract:**

Lidocaine exerts potential anti-tumor effects on various cancer cell lines, and its intravesical instillation is considered safer than intravenous administration for bladder cancer. However, the mechanisms underlying its anti-tumor effects have not been fully elucidated. Here, we aimed to elucidate the anti-tumor molecular mechanisms of lidocaine in bladder cancer cells and a xenograft model to substantiate the efficacy of its intravesical administration. We investigated the anti-proliferative and autophagyinducing activities of lidocaine in Nara Bladder Tumor No. 2 (NBT-II) rat bladder carcinoma cells using cell viability, flow cytometry, a wound healing assay, and western blotting. We also established a xenograft mouse model of bladder cancer, and cancer growth was examined using in vivo bioluminescence imaging. Lidocaine decreased cell viability, induced G0/G1 phase cell cycle arrest, and inhibited cell migration partially via glycogen synthase kinase (GSK) 3β phosphorylation. Moreover, a combination of lidocaine and SB216763 (a GSK3β inhibitor) suppressed autophagy-related protein expression. Bafilomycin-A1 with lidocaine significantly enhanced microtubule-associated protein 1A/1B-light chain (LC3B) expression; however, it decreased LC3B expression in combination with 3-methyladenine compared to lidocaine alone. In the xenograft mouse model, the bladder cancer volume was reduced by lidocaine. Overall, lidocaine exerts anti-proliferative effects on bladder cancer via an autophagy-inducing mechanism.

## 1. Introduction

Intravenous lidocaine infusion is being increasingly applied in clinical practice. Lidocaine reportedly inhibits inflammatory responses [1,2], exerts potential anti-tumor effects on various cancer cell lines [3,4,5], and enhances anti-cancer drug sensitivity [6]. However, only a few studies have addressed the molecular mechanisms underlying the anti-tumor effects of lidocaine. When lidocaine exceeds the appropriate blood concentration after intravenous administration, it can cause severe adverse effects, including convulsions, cardiac toxicity, and mortality [7], hampering its clinical application.

Bladder cancer is prone to frequent recurrence even in the early stages and has a poor prognosis, with a low 5-year survival rate, owing to uninhibited tumor cell proliferation, apoptosis evasion, and metastasis [8]. Conventional treatment approaches for bladder cancer include intravesical drug therapy, transurethral resection of bladder tumors, radical cysto-prostatectomy, and radiotherapy or chemotherapy, depending on the extent of muscle invasion by the cancer [9]. Consequently, postoperative bladder instillation of therapeutic agents is pivotal for removing residual cancer cells and reducing the recurrence rate in early-stage, non-muscle-invasive bladder cancer [10]. Generally, drugs such as pirarubicin, epirubicin, mitomycin C, hydroxycamptothecin, and Bacillus Calmette–Guérin are selected for intravesical administration [11,12]. However, a limitation of these drugs is their propensity to induce severe toxic and adverse reactions including chemical cystitis and cysto-spasm [13,14,15]. Other limitations include their narrow therapeutic efficacy, with recurrence rates as high as 30–50% within 2 years of drug use, and the substantial economic burden imposed on patients [9,16].

Intravesical lidocaine instillation has been introduced for treating interstitial cystitis/painful bladder syndrome [17,18]. Intravesical lidocaine administration thrice weekly for 2 weeks has been shown to provide immediate and sustained pain relief, as well as reduce symptoms related with a urinary incontinence in patients with interstitial cystitis/painful bladder syndrome [19]. In this study, we aimed to elucidate the anti-tumor effects of lidocaine on bladder cancer cells and a xenograft mouse model and the underlying molecular mechanisms to substantiate the efficacy of intravesical lidocaine instillation.

## 2. Materials and Methods

### 2.1. In Vitro Experiments

#### 2.1.1. Cell Culture

Nara Bladder Tumor No. 2 (NBT-II) cells were purchased from American Type Culture Collection (ATCC, Manassas, VA, USA) and cultured in Dulbecco’s modified Eagle medium (DMEM) supplemented with 100 U/mL penicillin, 100 µg/mL streptomycin, and 10% fetal bovine serum (FBS) at 37 °C in an environment of 5% CO_2_ and 95% humidity. DMEM and its supplements were acquired from Gibco (Carlsbad, CA, USA). Lidocaine was obtained from Sigma-Aldrich (St. Louis, MO, USA).

U0126 (a mitogen-activated protein kinase 1/2 inhibitor), SB216763 (a glycogen synthase kinase 3β (GSK3β) inhibitor), SP600125 (a c-Jun N-terminal kinase inhibitor), and Go6976 (a potent protein kinase C [PKC] inhibitor) were purchased from Cell Signaling Technology (Danvers, MA, USA). Bafilomycin A1 (Baf-A1) and 3-methyladenine (3-MA) were procured from Sigma-Aldrich.

#### 2.1.2. Cell Viability and Cell Cycle Analysis

For cell viability analysis, NBT-II cells (5 × 10^3^) were seeded in 96-well culture plates and incubated in the dark at room temperature (22 ± 2 °C) overnight. Thereafter, the cells were incubated in DMEM supplemented with 10% FBS and lidocaine at the concentrations indicated below for 2 days. The cells were then incubated with or without an inhibitor for another 1 or 2 days. Cell viability was analyzed using the EZ-Cytox Cell Viability Assay Kit (Dogen, Seoul, South Korea) according to the manufacturer’s instructions; the absorbance of the samples was measured at 450 nm using a VERSAmaxmicroplate reader (Molecular Devices, San Jose, CA, USA). The experiments were performed in triplicates with six biological replicates.

According to a previous study [20], cell cycle analysis was conducted with flow cytometry using propidium iodide (PI) staining. NBT-II cells were seeded in DMEM at a density of 3 × 10 cells/well in 60 mm Petri dishes for 24 h. Cells were cultured in a serum-free medium for 24 h and stained with PI. Thereafter, cells were treated with various concentrations (0, 0.05, 0.1, 0.5, and 1 mM) of lidocaine for 48 h, followed by a 3-(4,5-dimethylthiazol-2-yl)-2,5-diphenyl Tetrazolium bromide assay. Cells were dissociated by 0.25% trypsin-ethylene-diamine-tetra acetic acid (EDTA, Gibco) and fixed in 70% ethanol for 30 min at 4 °C. Subsequently, the cells were washed twice with cold phosphate-buffered saline (PBS) and treated with 0.25 μL of RNase (10 mg/mL) solution in 50 μL of PBS for 30 min at 37 °C. The cells were incubated with 5 μL of PI solution (Thermo Fisher Scientific, Waltham, MA, USA), and then 50 μL of PBS was added and mixed thoroughly. The cells were incubated for 30 min in the dark at room temperature (22 ± 2 °C), and the labeled cells were analyzed using a BD FACScan flow cytometer (Becton Dickinson Biosciences, Franklin Lakes, NJ, USA).

#### 2.1.3. Wound Healing Assay

Cells were seeded in 35 mm culture plates at a density of 2 × 10^4^ cells. Serum starvation was initiated 16 h prior to the experiment to prevent cell proliferation during migration. Confluent monolayers (80–90%) were artificially wounded by scratching with a plastic pipette tip and 1 mM lidocaine was added to the cells, which were then incubated for 36 h. The wounds were examined under a microscope, and images were captured after incubation. These experiments were repeated at least thrice.

#### 2.1.4. Western Blot Analysis

Cells were seeded in 60 mm tissue culture dishes and incubated for 24 h. To study the dose–response effect of lidocaine on cell cycle check-point protein expression and autophagy, the cells were treated with lidocaine (0.1, 0.25, 0.5, or 1 mM) with or without an inhibitor at 10 µM for 24 h. After washing the cells twice with ice-cold PBS, immunoblotting and total protein extraction were performed as previously described [21]. The primary antibodies included anti-phospho-GSK3β, anti-GSK3β, anti-ATG-5 and -7, anti-BECN1, anti-LC3B, anti-p27, anti-CCND1 (Cell Signaling Technology), and anti-glyceraldehyde-3-phosphate dehydrogenase (GAPDH; Invitrogen, Carlsbad, CA, USA).

#### 2.1.5. Real-Time Polymerase Chain Reaction

The expression levels of microtubule-associated protein 1A/1B-light chain (*LC3B*) and beclin-1 (*BECN-1*) were assessed using a real-time polymerase chain reaction (qPCR) with the SensiFAST SYBR Hi-ROX kit (Bioline USA, Taunton, MA, USA) and Applied Biosystems (AB) 7500 Fast qPCR System (Applied Biosystems, Foster City, CA, USA). This qPCR was performed four times. The target gene expression was normalized to the expression of the reference housekeeping gene GAPDH. The normalized cycle threshold of the normal sham group was used to calculate the fold difference for each group. We used several primers as follows; LC3B forward: 5′-CGGAGCTTCGAACAAAGAGT-3′, LC3B reverse: 5′-TCTCACCCTTGTATCGCTCT-3′, BECN-1 forward: 5′-TTCAAGACTTCGGACCGCGT-3′, BECN-1 reverse: 5′-AGTTAGCCTCTTCCTCCTGG-3′, GAPDH forward: 5′-CTGAGAATGGGAAGCTGGTC-3′, GAPDH reverse: 5′-CTCCACGACATACTCAGCAC-3′.

#### 2.1.6. Lentiviral Particle Transduction

NBT-II cells were cultured in DMEM supplemented with 100 U/mL penicillin, 100 µg/mL streptomycin, and 10% heat-inactivated FBS. Prior to viral infection, the cells (5 × 10^4^) were cultured in a 24-well plate and incubated overnight in 0.5 mL of a complete optimal medium with serum and antibiotics (Gibco, New York, MA, USA) for inducing NBT-II-Luc cells. The medium was replaced with 0.5 mL of the complete medium containing 6 μg/mL polybrene after 24 h. Cells were cultured overnight at room temperature (22 ± 2 °C) in the dark. Then, 50 μL of RediFect lentiviral particles (PerkinElmer, Waltham, MA, USA) were added to the culture to infect the cells. Stabilized clones of NBT-II-Luc cells were chosen using 5 μg/mL puromycin dihydrochloride, and transduction efficiency was confirmed by optical IVIS spectra (PerkinElmer).

### 2.2. In Vivo Study

#### 2.2.1. Xenograft Model

We employed seven-week-old male BALB/c nude mice (body weight 19–20 g) according to the Guide for the Care and Use of Laboratory Animals (National Institutes of Health). To establish the mouse xenograft model, 19 BALB/c nude mice were administered with NBT-II-Luc cells (2 × 10^6^ in 50 µL PBS) into the bladder. The mice were randomly assigned to either a saline- or lidocaine (100 mM/kg/day)-treated group to assess the response of bladder tumor xenografts.

Three weeks after xenograft modeling, tumor growth was confirmed using in vivo bioluminescence imaging. Lidocaine was instilled for 4 h into the bladders of BALB/c nude mice under isoflurane anesthesia; normal saline was administered to mice in the control group. This process was repeated thrice, with 2-day intervals between administrations. No procedure-related mortality of mice was observed. Two weeks after the lidocaine treatment, imaging was performed using the in vivo imaging system (IVIS); the mice were subsequently euthanized by isoflurane ~2–3%. All experiments were conducted in triplicates.

#### 2.2.2. In Vivo Bioluminescence Imaging (IVIS)

The xenograft model was employed to evaluate the effect of lidocaine on the growth of NBT-II-Luc cells. The xenografted bladder cancer mice were divided into two groups randomly with 7 mice per group: the saline injection group (control group, Con) and the lidocaine injection group (lidocaine group, Lido, 20 mM·day^−1^). To create bioluminescence signals, 150 mg/kg D-luciferin (potassium salt, PerkinElmer) was administered intraperitoneally for the bioluminescence imaging. Mice were anesthetized with isoflurane and placed in the imaging chamber. All fluorescence images were attained after 10 min of exposure. NBT images were obtained through the IVIS Spectrum and analyzed by Living Image 4.5.5 software (PerkinElmer). Regions of interest were marked on the tumor for quantitative comparisons, and the results were expressed as mean ± standard deviation.

#### 2.2.3. Assay of Autophagic Flux

NBT-II cells were cultured for 24 h with or without lidocaine treatment. We treated NBT-II cells using Baf-A1 (50 nM) 3 h before harvest for Western blot analysis to determine autophagic flux. Then, they were washed twice with cold PBS and dissolved in a radioimmunoprecipitation assay buffer containing protease inhibitors. Western blot analysis was conducted with anti-LC3B and anti-GAPDH antibodies.

### 2.3. Statistical Analysis

The data are expressed as mean ± standard deviation. Statistical significance was assessed using Student’s *t*-test or a one-way analysis of variance with Bonferroni correction. Statistical analyses were performed using the SAS version 9.4 (SAS Institute Inc., Cary, NC, USA) and Prism 5.01 software (GraphPad, San Diego, CA, USA). Results with *p* < 0.05 were considered statistically significant.

## 3. Results

### 3.1. NBT-II Cell Viability

**Figure 1:**
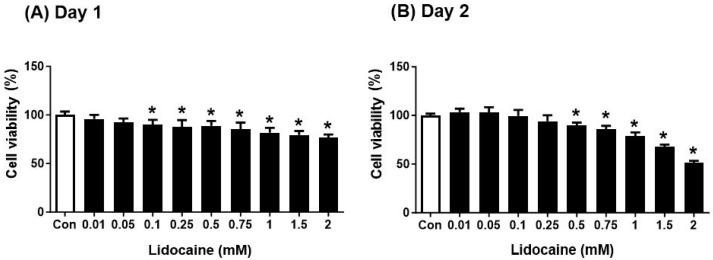
Lidocaine reduces NBT-II cell viability. Lidocaine reduced NBT-II cell proliferation, as determined using the MTT assay conducted at (**A**) 24 and (**B**) 48 h. Statistical significance was assessed using one-way analysis of variance with Bonferroni correction. * *p* < 0.05 compared to the saline-treated control group. NBT-II, Nara Bladder Tumor No. 2; MTT, 3-(4,5-dimethylthiazol-2-yl)-2,5-diphenyltetrazolium bromide.

NBT-II cells exposed to lidocaine (0.01–2 mM) for 2 days exhibited a reduction in viability compared to those in the control group (Figure 1).

### 3.2. Lidocaine Arrests NBT-II Cell Cycle in the G0/G1 Phase

**Figure 2:**
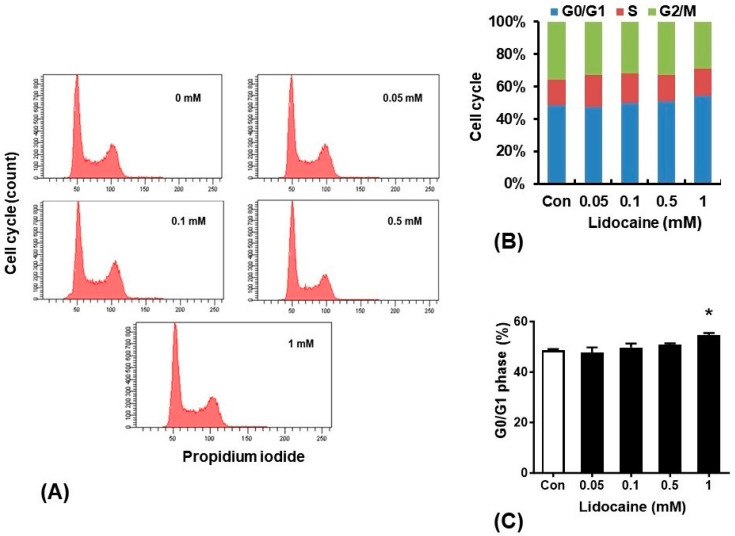
Lidocaine arrests NBT-II cell cycle in the G0/G1 phase. Flow cytometry analysis showed that lidocaine increased NBT-II cell cycle arrest, as determined using cell cycle assessment ((**A**), x-axis: propidium iodide, y-axis: count of cell cycle), quantification of each phase in the cell cycle ((**B**), x-axis: concentration of lidocaine, y-axis: percentage of each phase in cell cycle), and quantitation of the G0/G1 phase ((**C**), x-axis: concentration of lidocaine, y-axis: percentage of G0/G1 phase in cell cycle). Statistical significance was assessed using one-way analysis of variance with Bonferroni correction. * *p* < 0.05 compared to the saline-treated control group.

The cell cycle analysis revealed the inhibitory effect of lidocaine on NBT-II cell proliferation. Lidocaine treatment at concentrations of 0.05, 0.1, 0.5, and 1 mM for 24 h led to cell cycle arrest; specifically, 1 mM lidocaine-treated cells were arrested in the G0/G1 phase (Figure 2).

### 3.3. Lidocaine Suppresses NBT-II Cell Migration

**Figure 3:**
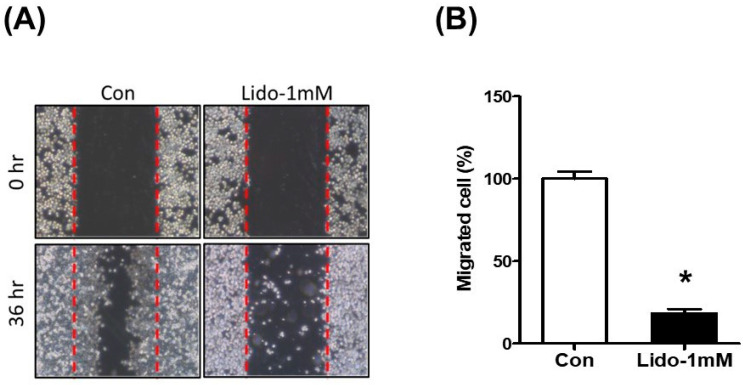
Lidocaine inhibits NBT-II cell migration. The wound healing assay (**A**) and quantitation of the migrated cells (**B**) demonstrated that lidocaine (1 mM) suppressed NBT-II cell migration. * *p* < 0.05 compared to the saline-treated control group.

The wound healing assay demonstrated a significant effect of lidocaine on NBT-II cell migration. The wound width in the control group decreased after 36 h as cells migrated into the wound. In contrast, lidocaine-treated cells exhibited minimal change in the wound width compared to cells in the control group (Figure 3).

### 3.4. Lidocaine Induces Changes in GSK3β Phosphorylation and Autophagy-Related Protein Expression

**Figure 4:**
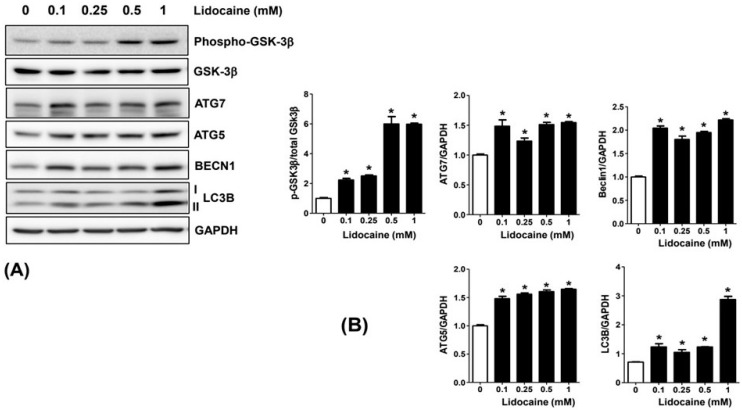
Lidocaine stimulates GSK3β phosphorylation and upregulates ATG5, ATG7, LC3B, and BECN1 expression (**A**). Western blot analysis showed that lidocaine increased GSK3β phosphorylation, as well as ATG5, ATG7, LC3B, and BECN1 expression in NBT-II cells relative to the saline-treated control (**B**). GAPDH was used for normalizing protein expression. Data was presented as the mean ± standard deviation (SD). * *p* < 0.05 compared to the saline-treated control group. GSK3β, glycogen synthesis kinase 3 beta; ATG5, autophagy-related 5; ATG7, autophagy-related 7; LC3B, microtubule-associated protein 1A/1B-light chain 3; BECN1, beclin-1; GAPDH, glyceraldehyde-3-phosphate dehydrogenase.

Lidocaine stimulated GSK3β phosphorylation in NBT-II cells. Compared to the control group, the group treated with lidocaine for 2 days in the concentration range of 0.1–1 mM showed increased GSK3β phosphorylation. GSK3β phosphorylation peaked at lidocaine concentrations of 0.5 and 1 mM. Additionally, compared to the control, lidocaine treatment elevated the levels of autophagy-related (ATG)3, ATG5, LC3B, and BECN-1 in NBT-II cells (Figure 4 and Figure S1).

### 3.5. Lidocaine Regulates the Protein Expression of p27, Cyclin D1, LC3B, and BECN1 via GSK3β Phosphorylation in NBT-II Cells

**Figure 5:**
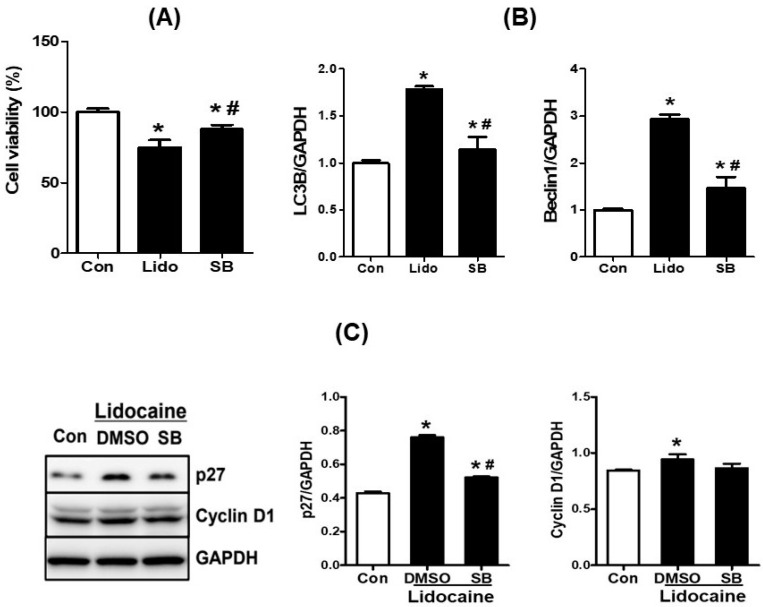
Lidocaine regulates the expression of p27, CCND1, LC3B, and BECN1 via GSK3β phosphorylation in NBT-II cells. (**A**) Cell viability analysis demonstrated that the co-treatment of cells with lidocaine and SB216763 abolished the effect of lidocaine on NBT-II cell viability. (**B**) The qPCR analysis revealed that the co-treatment of cells with lidocaine and SB216763 abolished the effect of lidocaine on the mRNA expression of *LC3B* and *BECN1*. (**C**) The Western blot analysis showed that lidocaine treatment increased the protein expression of p27 and cyclin D1 in NBT-II cells, whereas co-treatment with lidocaine and SB216763 decreased their expression. * *p* < 0.05 compared to the saline-treated control group. # *p* < 0.05 compared to the lidocaine-treated group. CCN1, cyclin 1; GSK3β, glycogen synthesis kinase 3 beta; SB216763 (SB), GSK3β inhibitor; qPCR, real-time polymerase chain reaction; Con, control.

To confirm whether lidocaine contributed to the reduction in cell proliferation and stimulation of autophagy-related protein expression in NBT-II cells through the GSK3β signaling pathway, we used a specific GSK3β inhibitor, SB216763. Compared to the control, lidocaine treatment reduced NBT-II cell proliferation, whereas co-treatment with lidocaine and SB216763 had no significant effect on NBT-II cell proliferation (Figure 5A). The mRNA level of *LC3B* increased after lidocaine treatment (LC3B/GAPDH ratio, 1.78 ± 0.03, *p* < 0.05); however, it was reduced following co-treatment with lidocaine and SB216763 (LC3B/GAPDH ratio, 1.14 ± 0.13, *p* < 0.05). A similar pattern was observed for *BECN1* mRNA expression (BECN1/GAPDH, 2.94 ± 0.09 vs. 1.47 ± 0.23, *p* < 0.05, respectively; Figure 5B). Western blotting demonstrated that the protein levels of p27 and cyclin D1 (CCND1), a cell cycle-specific marker, increased after lidocaine treatment but decreased following co-treatment with lidocaine and SB216763 (Figure 5C and Figure S2).

### 3.6. Intravesical Instillation of Lidocaine Decreases Bladder Cancer Development in a Xenograft Mouse Model Injected with NBT-II-Luc Cells

**Figure 6:**
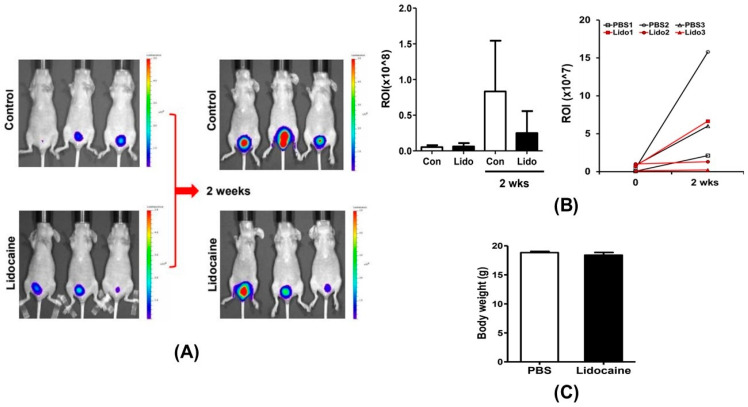
Lidocaine decreases tumorigenesis in mice bearing NBT-II-Luc tumors. In vivo imaging images (**A**) and individual data points in the bar graph (**B**) are demonstrated as ROI for these two groups, along with the tumor burden. There was no significant difference in body weight of the mice between the two groups (**C**). ROI, region of interest.

The volume of bladder tumors in nude mice intravesical treated with lidocaine decreased by approximately 50% compared with that in saline-treated nude mice, as evidenced in the IVIS analyses. The fluorescence intensity indicated an increasing tumor burden, ranging from low to high (blue to red shade), as quantified by the region of interest (Figure 6).

### 3.7. Lidocaine Regulates Autophagy in NBT-II Cells

**Figure 7:**
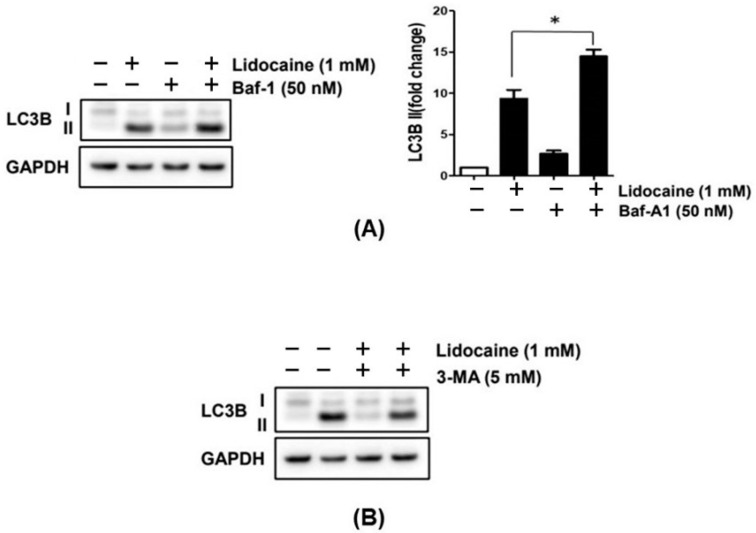
Lidocaine induces autophagy in NBT-II cells. (**A**) Co-treatment of NBT-II cells with lidocaine and Baf-1 increased LC3B expression to a greater extent than lidocaine alone, as evidenced in the Western blot analysis. (**B**) Lidocaine treatment of NBT-II cells increased the protein expression of LC3B, whereas co-treatment with lidocaine and 3-MA decreased its expression, as determined using the Western blot analysis. Baf-1, bafilomycin A1; 3-MA, 3-methyladenine. * *p* < 0.05 compared to the saline-treated control group.

The LC3B protein level increased in the lidocaine-treated NBT-II cells compared with that in the control group. The addition of Baf-A1 (50 nM) to the lidocaine-treated NBT-II cells significantly increased LC3B expression compared with that in the lidocaine-treated NBT-II cells without Baf-A1, suggesting that lidocaine may act as an autophagy inducer in NBT-II cells (Figure 7 and Figure S3). The lidocaine treatment of NBT-II cells increased the protein expression levels of LC3B, whereas co-treatment with lidocaine and 3-MA decreased them in the Western blot analysis.

## 4. Discussion

Our in vitro experiments showed a time-dependent decrease in the viability of NBT-II cells following lidocaine administration. This decrease was accompanied by cell cycle arrest in the G0/G1 phase and reduced cell migration; these effects were noticeable even at lidocaine concentrations lower than 0.23 mg/mL. Lidocaine also increased LC3B expression in NBT-II cells, confirming its potency as an autophagy inducer; LC3B expression increased more significantly upon co-treatment with lidocaine and Baf-A1 than with treatment with lidocaine alone. This induction of autophagy was associated with the activation of the GSK3β signaling pathway. Additionally, intravesical instillation of 0.05% lidocaine (2 mM) attenuated bladder cancer growth in xenograft nude mice injected with NBT-II-Luc cells. To the best of our knowledge, this is the first study to confirm that bladder tumor growth can be attenuated even with intravesical administration of lidocaine as a single agent, and that the mechanism involves autophagy influx.

Lidocaine exhibits potential anti-tumor effects through various mechanisms, including direct effects on cell growth, apoptosis induction, and proliferation inhibition. It also indirectly affects natural killer cell activity [22], regulates the local microenvironment by modulating pro-inflammatory macrophages, and transforms mesenchymal stromal cells. It also decreases the anchorage-independent growth of breast cancer cells, revealing that the intraperitoneal administration of lidocaine reduces tumor progression and enhances the survival of mice with peritoneal carcinomatosis [23]. Furthermore, lidocaine inhibits tumor growth and induces apoptosis in human hepatic cells, and it suppresses the growth and metastasis of hepatocellular carcinoma (HCC) and sensitizing HCC cells to anti-cancer drugs in vivo [24]. In addition, lidocaine demonstrates anti-tumor effects in gastric cancer by inhibiting cell proliferation, migration, and invasion [25]; it inhibits the malignant development of cervical cancer by suppressing cancer cell proliferation and apoptosis [26]. Furthermore, lidocaine may inhibit human bladder cancer cell proliferation [27]. Yang et al. reported that lidocaine (at concentrations of 1.25–5 mg/mL) dose-dependently inhibited the proliferation of BIU 87 bladder cancer cells, and when administered in combination with anti-cancer agents, its anti-proliferative effects were enhanced [28].

GSK3 inhibitors simultaneously activate the autophagy and lysosomal networks [29,30,31]. Autophagy induces apoptosis in irreversibly non-viable tumor cells [32]. This self-degradation system plays a crucial role in maintaining cellular homeostasis under stress conditions and is implicated in the progression of numerous diseases [33]. Autophagy is also critical in cell survival and maintenance; it regulates the degradation of organelles, proteins, and macromolecules, as well as the recycling of the degradation products [34]. However, autophagy is a controversial pathway in human cancer cells, as it can act as a mechanism of both self-protection and apoptosis [35]. Therefore, factors that influence the condition-dependent behavior of autophagy should be investigated, and genes and signaling pathways should be identified to better understand the effect of autophagy on bladder cancer.

Our qPCR results demonstrated that lidocaine regulated the expression of p27 and LC3B via the GSK3β signaling pathway in NBT-II cells. The p27 was increased by lidocaine administration and decreased by GSK3β inhibitor treatment, significantly. Conversely, CCND1 seemed to be increased by lidocaine, but there was no significant change by the GSK3 β inhibitor. This is consistent with the aforementioned cell cycle showing arrest in the G1 phase induced by p27 [36]. The expression of LC3B increased after treatment with Baf-A1 and more significantly increased combined with lidocaine; but, it decreased after treatment with 3-MA. Baf-A1 disrupts autophagic flux by inhibiting vacuolar-type ATPase-dependent acidification and calcium-ATPase at 60A/sarco-endoplasmic reticulum calcium-ATPase-dependent autophagosome–lysosome fusion [37]. Accordingly, Baf-A1 was employed as an inhibitor of autophagosome–lysosome fusion, resulting in the continuous accumulation of autophagosomes, while 3-MA was considered as an early-stage inhibitor of autophagy [38]. Both have been used to investigate the effects of lidocaine on autophagy. The significant increase in LC3B caused by lidocaine does not indicate absolutely that it affected the generation of autolysosomes, which are created by autophagosomes combining with lysosomes during the autophagic flux [39]. Further research is also needed to identify the exact stage of autophagy at which lidocaine acts. However, it is clear that lidocaine caused a distinguished increase in LC3B, a remarkable protein related to autophagy [40]; hence, lidocaine can be recommended as an autophagy inducer in bladder cancer.

To determine the optimal dose [41,42] and mode of lidocaine administration [43] in our preclinical cancer model, we adapted protocols from clinical practice. Reagan-Shaw conducted a preclinical study and reported that a lidocaine dose of 100 mg/kg in mice corresponds to 8 mg/kg in humans, considering body surface area-based dose translation [42]. The intraperitoneal administration of 400 mg lidocaine (equivalent to 8 mg/kg in a 50 kg patient) is considered safe [44]. We injected approximately 0.5 mL of lidocaine (2 mM) into the bladders of mice, followed by three washes at 48 h intervals. This intravesical lidocaine administration reduced the bladder tumor burden in the xenograft mouse model without adverse effects, implying that intravesical lidocaine could be an alternative to the current intravesical therapy (without altering the blood concentrations) in the adjuvant treatment after endoscopic transurethral resection of the bladder tumor for non-muscle-invasive bladder cancer.

Our translational study has certain limitations. Our in vitro cell-based experiments were conducted with a single cell line (NBT-II cells), except the cell viability using 253 J cells (human derived bladder cancer cell line) (Figure S4). To confirm the anti-tumor effects of lidocaine in bladder cancer cells, further investigations in other cell lines are warranted. Furthermore, considering that this research was performed using a small laboratory animal, prospective human studies are necessary to establish a definite anti-tumor effect of lidocaine and develop a protocol for intravesical lidocaine administration in patients with bladder cancer. Nevertheless, our findings revealed an association among lidocaine, autophagy, and bladder cancer. Lidocaine, as a robust autophagy inducer, inhibits the growth of bladder cancer cells through GSK3β signaling. We demonstrated the potential of intravesical lidocaine administration in reducing the bladder tumor burden with no adverse effects in a xenograft mouse model.

## 5. Conclusions

We established an association between the molecular mechanism of tumor development and lidocaine action through in vitro experiments. Additionally, in vivo experiments demonstrated the safety of intravesical lidocaine administration at appropriate concentrations, application times, and frequencies. These findings highlight the potential of intravesical lidocaine injection for clinical application. Further successful validation of the effectiveness of intravesical lidocaine administration in patients with bladder cancer in clinical trials could help establish this drug as a novel adjuvant treatment for bladder cancer. We believe that our findings can help establish a clinically applicable protocol for the application of lidocaine as an adjuvant therapy post-bladder cancer surgery.

## Data Availability

The datasets used or analyzed in the current study are available from the corresponding authors on reasonable request.

## Supplementary Materials

The following supporting information can be downloaded at: https://www.mdpi.com/article/10.3390/cancers16071267/s1. Figure S1. Western blot analysis. GSK3β, glycogen synthesis kinase 3 beta; ATG5, autophagy-related 5; ATG7, autophagy-related 7; LC3B, microtubule-associated protein 1A/1B-light chain 3; BECN1, beclin-1; GAPDH, glyceraldehyde-3-phosphate dehydrogenase. Figure S2. Western blot analysis. Con, control; DMSO, dimethyl Sulfoxide; SB216763 (SB), GSK3β inhibitor; GAPDH, glyceraldehyde-3-phosphate dehydrogenase. Figure S3. Western blot analysis. Baf-1, bafilomycin A1; LC3B, microtubule-associated protein 1A/1B-light chain 3; 3-MA, 3-methyladenine; GAPDH, glyceraldehyde-3-phosphate dehydrogenase. Figure S4. Lidocaine reduces 253 J cell viability. Lidocaine reduced 253 J cell proliferation, as determined using the MTT assay conducted at (A) 24 and (B) 48 h. Statistical significance was assessed using one-way analysis of variance with Bonferroni correction. * *p* < 0.05 compared to the saline-treated control group. Amount of 253 J, human derived bladder cancer cell line; MTT, 3-(4,5-dimethylthiazol-2-yl)-2,5-diphenyltetrazolium bromide.
