# Supplementary material for: Anti-Proliferative Effects of Lidocaine as an Autophagy Inducer in Bladder Cancer via Intravesical Instillation: In Vitro and Xenograft Mouse Model Experiments
Source: Cancers (Basel). 2024 Mar 24;16(7):1267. doi: 10.3390/cancers16071267 (PMC11010986; doi:10.3390/cancers16071267)
Supplement: Supplementary file 1 [file cancers-16-01267-s001.zip › cancers-2885998-supplementary.pdf]

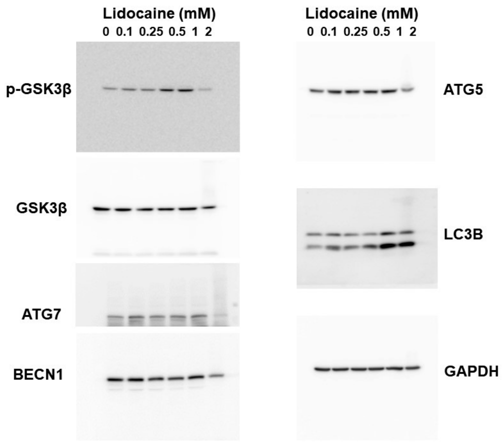

Figure S1. Western blot analysis. GSK3β, glycogen synthesis kinase 3 beta; ATG5, autophagy-related 5; ATG7, autophagy-related 7; LC3B, microtubule-associated protein 1A/1B-light chain 3; BECN1, beclin-1; GAPDH, glyceraldehyde-3-phosphate dehydrogenase.

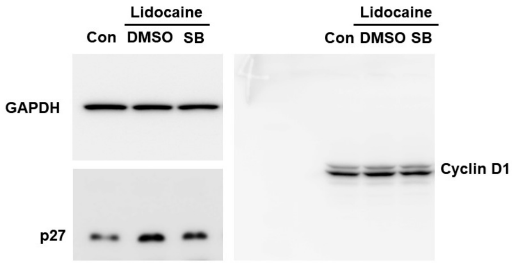

Figure S2. Western blot analysis. Con, control; DMSO, dimethyl Sulfoxide; SB216763 (SB), GSK3β inhibitor; GAPDH, glyceraldehyde-3-phosphate dehydrogenase.

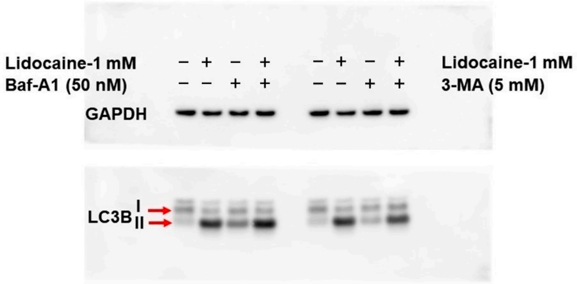

Figure S3. Western blot analysis. Baf-1, bafilomycin A1; LC3B, microtubule-associated protein 1A/1B-light chain 3; 3-MA, 3-methyladenine; GAPDH, glyceraldehyde-3-phosphate dehydrogenase.

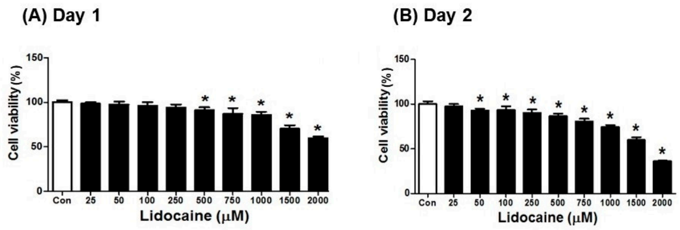

Figure S4. Lidocaine reduces 253 J cell viability. Lidocaine reduced 253 J cell proliferation, as determined using the MTT assay conducted at (**A**) 24 and (**B**) 48 h. Statistical significance was assessed using one-way analysis of variance with Bonferroni correction. * $p < 0.05$ compared to the saline-treated control group. Amount of 253 J, human derived bladder cancer cell line; MTT, 3-(4,5-dimethylthiazol-2-yl)-2,5-diphenyltetrazolium bromide.
